# Design and evaluation of collaborative decision‐making application for patient care in the emergency department

**DOI:** 10.1002/hsr2.1931

**Published:** 2024-02-25

**Authors:** Neda Shams‐Vahdati, Samad Shams Vahdati, Taha Samad‐Soltani

**Affiliations:** ^1^ Department of Health Information Technology, School of Management and Medical Informatics Tabriz University of Medical Sciences Tabriz Iran; ^2^ Emergency and Trauma Care Research Center Tabriz University of Medical Sciences Tabriz Iran

**Keywords:** collaboration, emergency department, informatics, messaging app, smartphone

## Abstract

**Background and Aims:**

Collaboration has become a crucial element of effective healthcare delivery in the emergency department (ED). In high‐pressure environments, healthcare providers can prioritize patients by consulting with other specialists to gain diverse perspectives and arrive at a shared understanding of the best course of action. It was conducted for the purpose of designing and evaluating the collaborative decision‐making application for patient care in the ED.

**Methods:**

The present applied research study was conducted between April 1, 2021 and May 31, 2023 at Imam Reza Hospital of Tabriz University of Medical Sciences. The study was conducted in three phases: exploration, development, and evaluation, utilizing modern technologies such as Flutter and Node.js to design and program the application. The effectiveness of the system was evaluated using established measures, including the think‐aloud protocol, user experience questionnaire, and Likert‐scale questionnaires developed by Ghadri et al.

**Results:**

The average scale for attractiveness was 2.03, perspicuity was 2.90, efficiency was 2.40, dependability was 1.93, stimulation was 2.48, and novelty was 2.78. Additionally, 71% of physicians gave a very good rating to the accessibility of necessary information at any time, motivation to use the system for accessing information, usefulness of the system compared to the time spent using it throughout the day. Furthermore, 57% of physicians gave a very positive rating to sharing information and knowledge, ease of using the search function and accessing the system, user control and monitoring, free access to the system, and support from colleagues and system users.

**Conclusion:**

The study suggests that introducing Information and Communication Technology such as medical apps can improve healthcare delivery by streamlining patient care, promoting effective teamwork, and reducing medical errors and treatment delays.

## 1 INTRODUCTION

The emergency department (ED) of a hospital is a multispecialty environment with high complexity and workload, critical, time‐sensitive, stressful, and unpredictable conditions. Therefore, during a patient's treatment in the ED, the available patient history and medical records may be incomplete or inaccurate, leaving clinicians with limited information to make fully informed treatment decisions. In this situation, choosing the most appropriate course of action can be difficult and sometimes impossible, leading to risks of communication failures due to incorrect information transfer that endangers patient safety. Ensuring successful and effective information exchange among medical professionals in the ED is highly significant.^1^, ^2^, ^3^, ^4^, ^5^, ^6^

Collaboration has become a crucial element of effective healthcare delivery, improving teamwork and reducing clinical error rates. Lack of communication is a significant barrier to collaboration, which is essential for a team to function cohesively. Collaboration among healthcare professionals has been shown to improve patient outcomes while reducing healthcare costs and increasing physician job satisfaction. Poor communication among healthcare professionals can lead to conflicts, medical errors, and poor patient outcomes. Therefore, fostering collaboration, teamwork, and team spirit are crucial elements in delivering high‐quality healthcare. The American Medical Association recognizes teamwork as a clinical competency.^7^, ^8^, ^9^, ^10^, ^11^, ^12^

Collaborative decision‐making is a process in which multiple individuals work together to make decisions about a particular project, issue, or specific decision. This approach entails sharing information and considering the strengths and weaknesses of various options, resulting in more responsive and effective solutions. Within healthcare, particularly EDs, collaborative decision‐making can play a crucial role in improving patient flow and enhancing service quality. In environments with high workload, healthcare providers can prioritize patients by consulting with other specialists to gain diverse perspectives and arrive at a shared understanding of the best course of action.^13^, ^14^, ^15^

To facilitate effective collaborative decision‐making, the widespread adoption of Information and Communication Technologies (ICTs) has been instrumental. ICTs refer to a range of technologies used for gathering, storing, retrieving, processing, transmitting, and displaying information electronically. In recent years, the widespread adoption of ICTs has led to an increase in the use of social networking platforms and messaging applications as tools for strengthening interconnected communication chains. These technologies offer great potential, accessibility, documentation, sustainable storage capacity, and extensive public use, making them valuable resources for supporting individual and group activities. Furthermore, smartphones are increasingly being utilized to support health informatics projects and public healthcare initiatives, and the healthcare industry is not exempt from this trend.^16^, ^17^, ^18^, ^19^

In Iran, the healthcare sector has increasingly adopted medical applications due to the inadequacy of traditional information systems in meeting diverse healthcare needs. Medical apps provide an easy‐to‐use interface that efficiently manages patient data and healthcare units, resulting in optimal utilization of time and resources. These apps offer healthcare professionals secure access to patient information, enabling them to deliver more efficient and effective care. As such, health informatics and m‐health applications have become increasingly important in enhancing healthcare delivery.^20^, ^21^, ^22^, ^23^, ^24^

This research paper introduces a novel decision‐making application created to improve collaboration and communication among healthcare providers in ED. The CDM application includes features such as patient admissions, creating profiles for physicians group chats, image/video sharing, access to medical forms and information, and consultation, providing a well‐structured framework for collaborative care. With easy access to patient data, specialists can quickly make informed diagnoses, and consultations with other healthcare professionals can be conveniently conducted through these apps. By promoting effective teamwork and communication among healthcare practitioners, the use of the CDM app enhances patient care while reducing waiting times, costs, and delays in treatment. This study aimed to prepare an application for communication in ED and compare it with other applications.

## 2 METHODS

**Figure 1:**
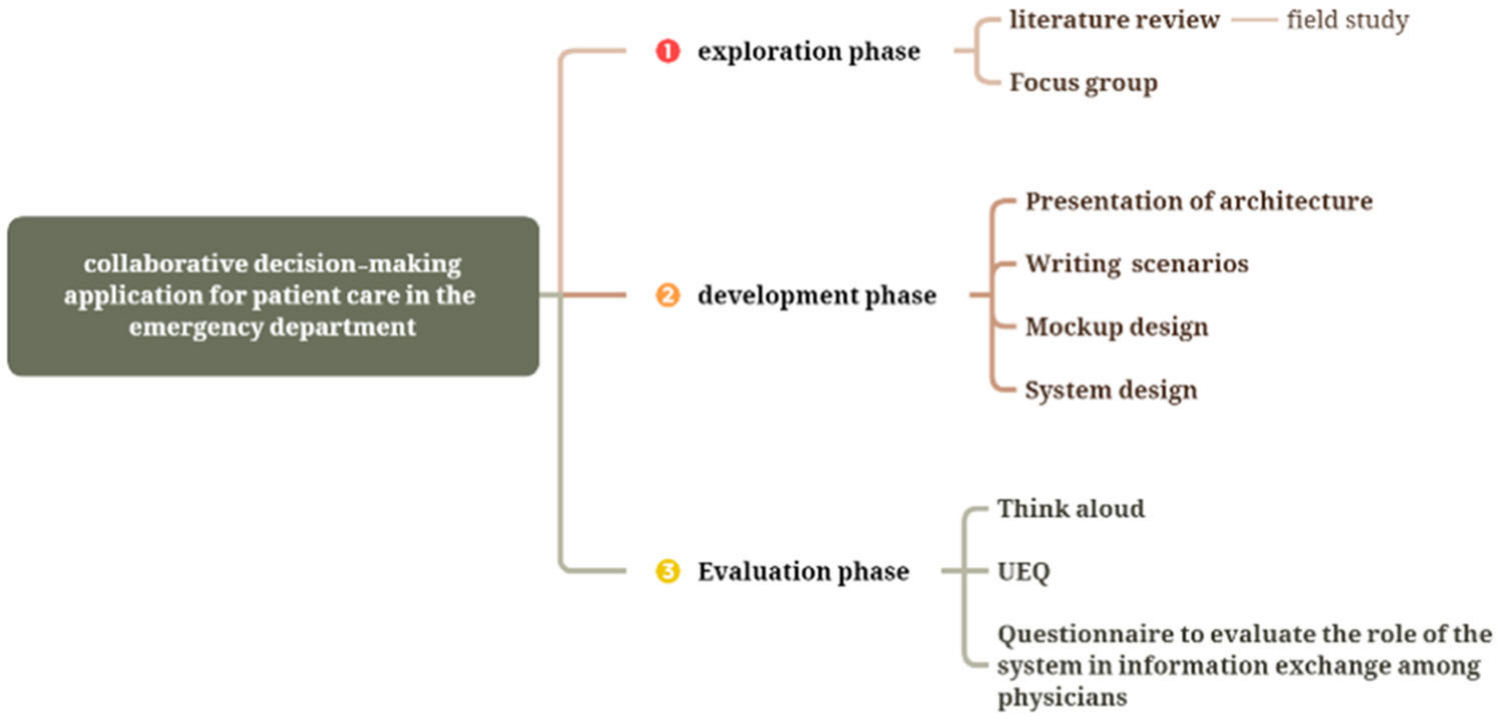
CDM was conducted in three exploratory, developmental, and evaluative phases. UEQ, user experience questionnaire.

The present applied research study was conducted between April 1, 2021 and May 31, 2023. The research comprised three distinct phases: exploration, development, and evaluation, as shown in Figure 1.

### 2.1 Exploration phase: Requirement analysis and data elicitation

During the exploration phase, the study relied on literature review and field study methodologies to extract the requirements of the ED of Imam Reza Hospital of Tabriz University of Medical Sciences, to develop a CDM app for emergency patient care. Scientific databases such as PubMed, Google Scholar, Scopus, and Google Search were searched to gather relevant studies and projects. The collected resources were then analyzed to extract both functional and nonfunctional requirements. Finally, two 40 min focus groups meetings were conducted with three experienced emergency medicine specialists to elicit and validate potential system requirements, resulting in a list of essential and feasible features. In addition, the organizational requirements of the hospital were also taken into consideration during the exploration phase.

In our study, we presented the results of our requirements engineering based on relevant articles to emergency medicine specialists at Imam Reza Hospital, with a sample size of five experts aged between 35 and 50. This allowed us to obtain valuable input from a specific group of experts who are familiar with the setting where the CDM app would be used. Through real‐time information sharing among healthcare professionals, we identified key features important for the development of the CDM app, including a triage page, real‐time video chat, personal chat, message editing, email registration, and more. We also found that stakeholders' biggest expectation of the CDM app is to have up‐to‐date and maintained information. During our focus group sessions, we conducted interviews with physicians and analyzed hospital infrastructure requirements, while considering cost factors, to determine additional features such as the ability to admit new patients, create patient groups, propose and send consultations in groups, and share various types of messages and files.

### 2.2 Development phase: System design and coding

The proposed architecture for the collaborative decision‐making application for patient care in the ED is based on a 3‐tier client‐server architecture. The server component comprises a management panel, a database, and an information exchange program. For better comprehension of the system and identification of relationships between its various components, we used Visual Paradigm to design and model UML diagrams. The development phase involved a presentation of the application's architecture and the design and programming procedures. The design process involved the development of use‐case scenarios and the designing and prototyping of an interface for the application using Figma.^25^ This approach allowed for necessary stakeholder feedback to be incorporated before proceeding with coding and programming then, the application was programmed using Flutter^26^ framework and Node.js^27^ language, with a NoSQL MongoDB^28^ database used to store user data in JSON^29^ format. The HTTP protocol^30^ links the user and the server. Real‐time communication between server and client was facilitated using the WebSocket protocol,^31^ and secure authentication and verification of user information were ensured using JWT^32^ tokens. The use of these technologies and methodologies allowed for optimal performance and improved user experience. The CDM app features a login page, account management for admin, physician profiles, admitting and viewing patient lists, patient information including name, code, age, gender, date, and time, creating patient groups, sending messages (text, voice, file, form, stickers, photo, video), as well as discharging and consulting patients.

### 2.3 Evaluation phase: Implementation and user experience evaluation

In the evaluation phase, participants evaluated the system in real‐world work environments with adequate lighting, a desk, two chairs, and a computer system. During the evaluation phase of the study, a pilot program was implemented in the ED with 500 patients for 1 month. The evaluation team consisted of two IT healthcare technology experts and two emergency physicians who used a think‐aloud protocol to verbalize their thoughts while performing assigned tasks. This approach aimed to provide a deeper understanding of the evaluators' mental models and decision‐making processes. The sessions were recorded using Screen Recorder,^33^ which captured user interactions with the application on mobile phones. The think‐aloud model was used to evaluate the flow of the application, including user login, account updating, acceptance of new patients, creation of patient groups, patient discharge, and proposing consultations by tagging physicians. Expert satisfaction was assessed using the user experience questionnaire (UEQ)^34^ questionnaire, which measures six dimensions of user experience: Attractiveness, perspicuity, efficiency, dependability, stimulation, and novelty. Scores on all six scales were averaged and compared to a benchmark, and graphs were created to visualize the results. UEQ consists of 26 items that are designed to capture the different dimensions of user experience. These dimensions are evaluated using bipolar adjectives, such as “attractive versus unattractive” or “stimulating versus boring,” on a scale ranging from 1 (strongly disagree) to 7 (strongly agree). Researchers often employ the UEQ in user studies to gather empirical data on users' perceptions and to compare user experiences across different systems or design variations.

Emergency medicine specialists completed a separate 42‐question Likert‐scale questionnaire to assess the system's effectiveness in improving CDM properties, feedback, accessibility, communication, time management, motivation, and task management.^35^ Statistical analysis was performed for each question and some measures including frequency (percentage) were calculated and reported.

## 3 RESULTS

### 3.1 System quality attributes

#### 3.1.1 Security

The application prioritizes the protection of information and system resources against unauthorized access, data breaches, and malicious activities. Robust measures such as authentication, encryption, access control, and vulnerability management have been implemented. The CDM application is not publicly available for download or sharing; rather, it is exclusively distributed and executed under the administration's supervision. Users are assigned a unique username and password, which are authenticated upon login. Access to the application is limited to the university's internal network, with external access facilitated through a secure VPN connection.

#### 3.1.2 Reliability

The application demonstrates a high level of reliability, ensuring consistent and error‐free performance. It reliably delivers accurate results and operates as expected without unexpected failures or errors.

#### 3.1.3 Performance

The application's performance is carefully optimized to provide a seamless user experience. It quickly responds to user interactions and effectively handles demanding workloads. Key performance indicators, such as response time, throughput, scalability, and resource utilization, have been rigorously assessed and refined.

#### 3.1.4 Usability

The application's user interface is designed with a focus on usability, aiming to enhance user efficiency and satisfaction. Considerable emphasis has been placed on ensuring that users can easily accomplish their tasks and navigate through the system. Aspects such as learnability, efficiency, and overall user satisfaction have been taken into account during the design and development process.

#### 3.1.5 Availability

The application aims to maintain a high level of availability, ensuring uninterrupted access for users. Downtime is minimized through the implementation of redundancy, fault tolerance, backup, and recovery mechanisms. These measures ensure that the system remains operational and accessible, maximizing uptime and minimizing any potential disruptions.

By prioritizing these system quality attributes, the application provides a secure, reliable, high‐performing, user‐friendly, and highly available platform for users to interact with.

### 3.2 Exploration phase

**Figure 2:**
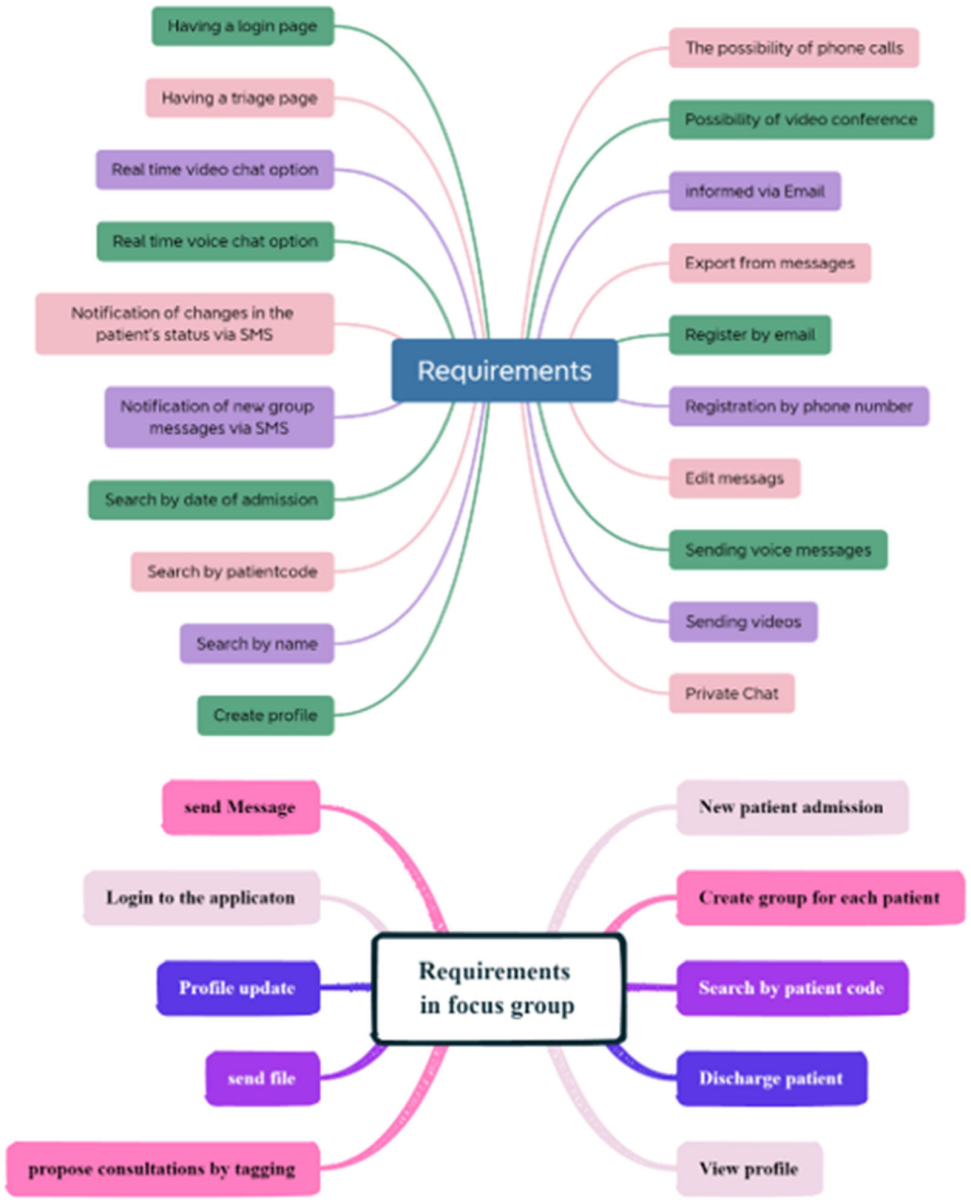
The list of requirements discussed and agreed upon in the focus group.

We conducted a review of the utility of focus groups for exploratory research and found them to be an effective approach. Preprepared forms, such as patient history, patient lists, and a search function to find patients by code, were also included (see Figure 2). Given the critical importance of prompt decision‐making in ED settings, we prioritized providing easily accessible, real‐time information within the ED.

### 3.3 Development phase

**Figure 3:**
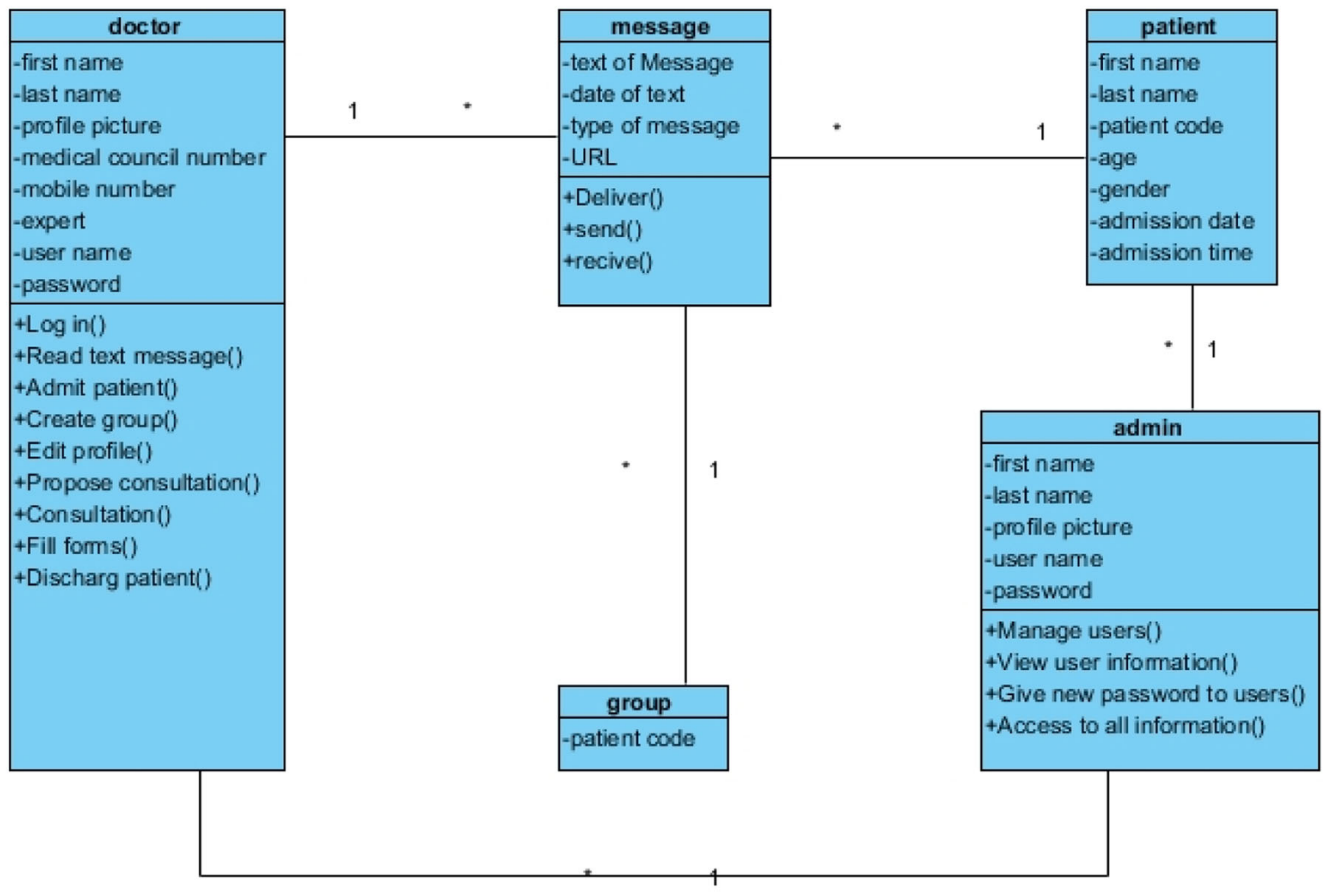
Class diagram of CDM.

For better comprehension of the system and identification of relationships between its various components, we used Visual Paradigm to design and model UML diagrams, including class diagrams and use‐case diagrams (Supporting Information: Appendice 1). The CDM classes include physician, admin, group, patient, and message objects (Figure 3).

**Figure 4:**
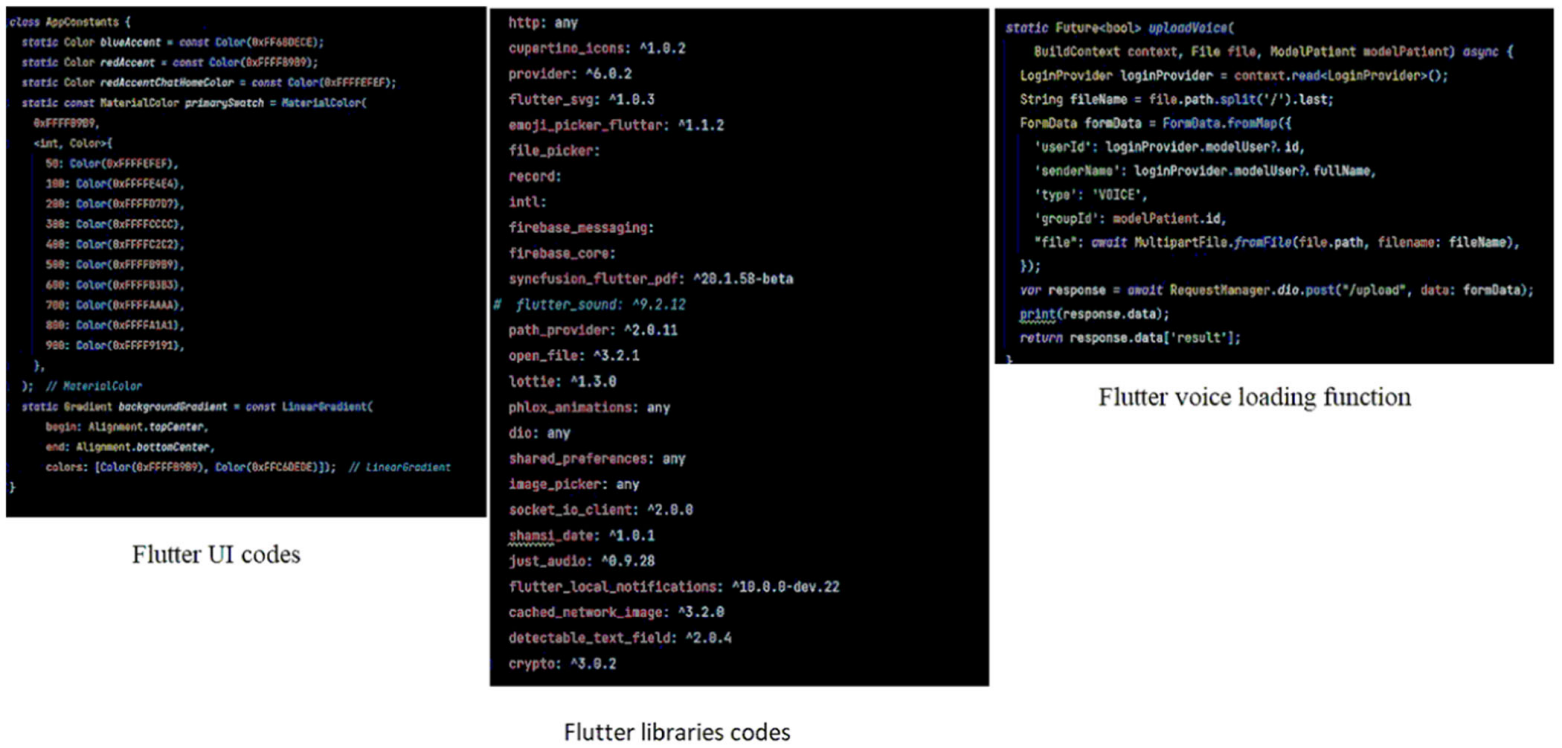
Flutter libaries codes.

We have scenarios for all use cases, including the one depicted in Supporting Information: Appendice 2 where we create a group for each patient in CDM APP. We designed icons for the CDM app, including team, a message sign, and an ED logo (shown in Supporting Information: Appendice 3). To design the application prototype, we used Figma to prototype (Supporting Information: Appendice 4) and Flutter to develop the app (Figure 4).

For the backend, we utilized Node.js and MongoDB to store user data (Supporting Information: Appendices 5,6). We also implemented the WebSocket protocol for real‐time communication between client and server and JWT as a security token for authentication and verification during interactions.

**Figure 5:**
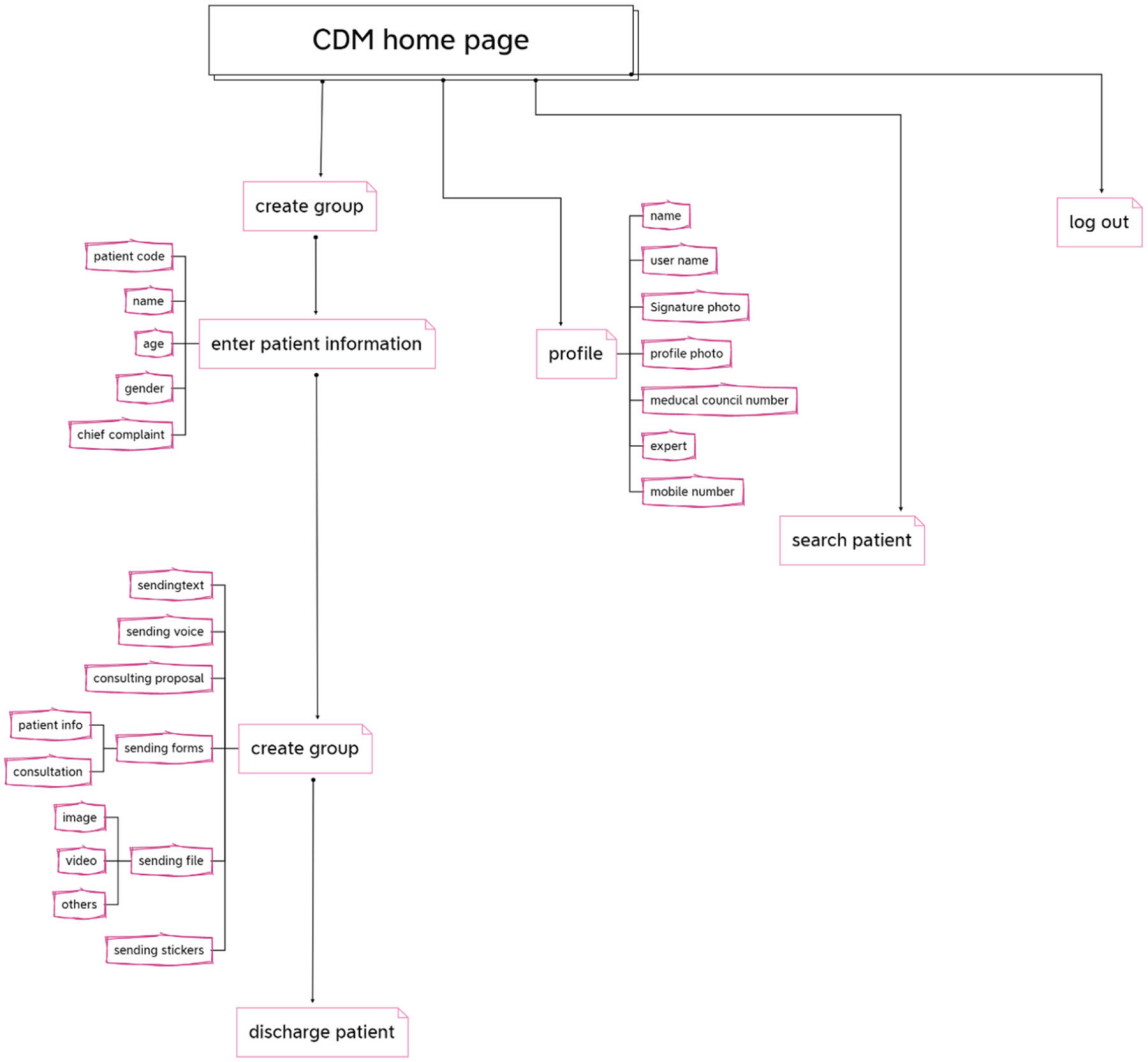
CDM app flow.

We create a streamlined user interface with a clear app flow (Figure 5).

**Figure 6:**
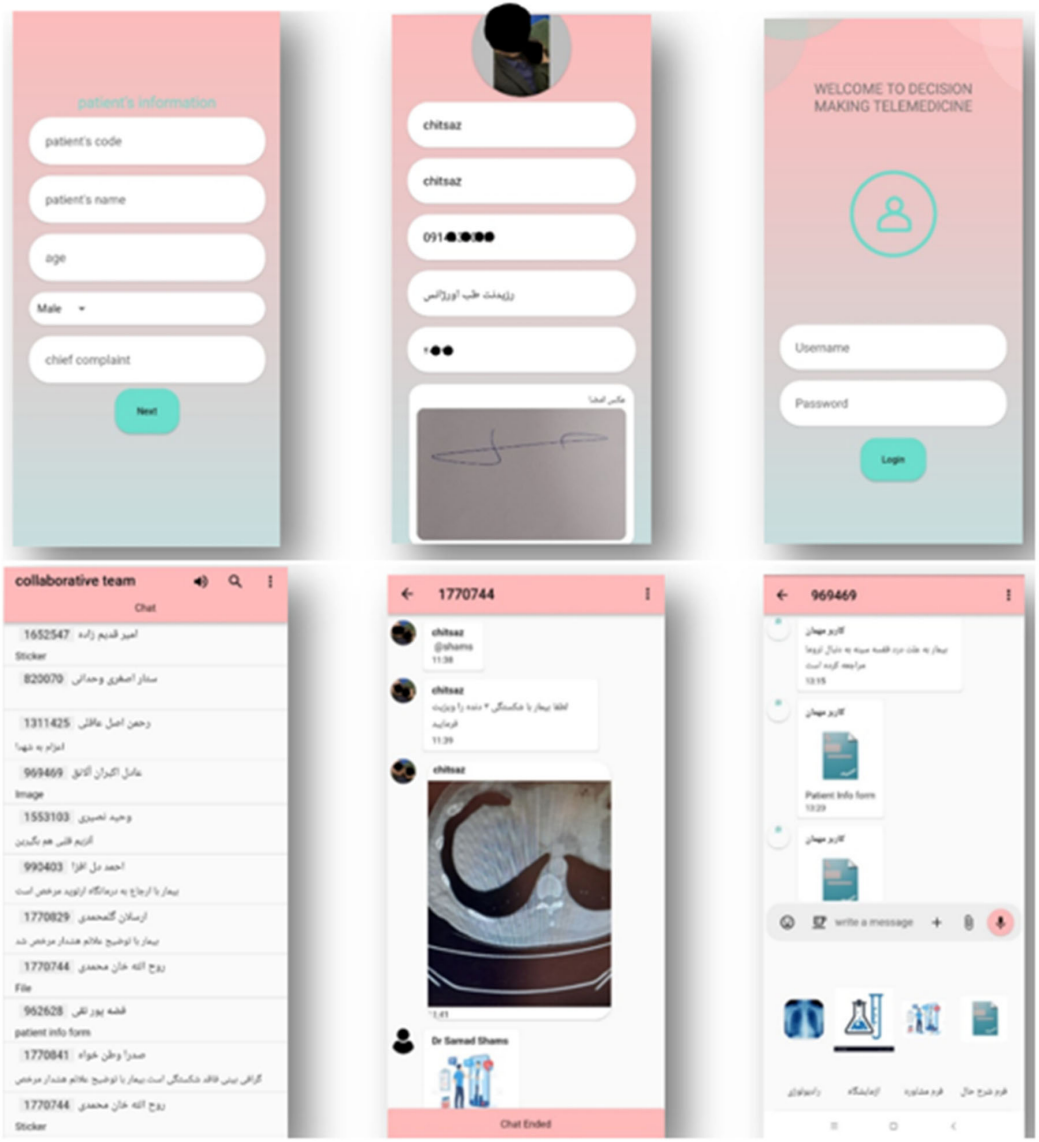
UI of CDM.

Physicians can edit patient profiles and send messages in created groups. Figure 6 in our study depicts the CDM app user interface (Supporting Information: Appendice 7).

The admin provides users with a username and password, and new passwords can be provided if necessary. Physicians can create groups for patients and edit their profiles. They can send text, voice, and file messages in the created groups.

### 3.4 Evaluation phase

During the evaluation phase of the study, a pilot program was implemented in the ED (Supporting Information: Appendice 8). Valuable feedback and suggestions were provided by users in a think‐aloud session to improve the system. Incorporating patient gender into admission procedures and adding date and time to all messages and groups were among the suggestions. The UEQ was used to measure user experience, and 10 respondents filled out the questionnaire. The analysis showed that the mean score for all scales ranged between 2 and 3, which falls within the excellent range, placing it among the top 10% of UEQ evaluations. Specifically, the average scale for attractiveness was 2.03, perspicuity was 2.90, efficiency was 2.40, dependability was 1.93, stimulation was 2.48, and novelty was 2.78. The quality of the CDM application is included in the “excellent” category compared to 468 different products in the benchmark data set (Supporting Information: Appendice 9). Table 1 groups CDM application users' interests by pragmatic quality (perspicuity, efficiency, dependability) and hedonic quality (stimulation and novelty), showing that stimulation has an average score of 2.48, while novelty has 2.78. Respondents' responses related to dependability stated that the CDM application is straightforward and trusted; it also provides guaranteed results and is very helpful in real‐time sharing. Despite the high novelty evaluation (2.78), after using the CDM application, they felt it was beneficial and exciting to use. This shows that the application can be accepted by the target users of this application to help interactions. The survey results indicated that physicians who used the program were well‐received, with all physicians rating their motivation for using the system as the highest score due to the possibility of achieving better patient outcomes.

**Table 1:** Pragmatic and hedonic qualities of the CDM application (*n* = 10).

Scale	Mean	Comparison to benchmark	Interpretation
Attractiveness	2.03	Excellent	In the range of the 10% best results
Perspicuity	2.90	Excellent	In the range of the 10% best results
Efficiency	2.40	Excellent	In the range of the 10% best results
Dependability	1.93	Excellent	In the range of the 10% best results
Stimulation	2.48	Excellent	In the range of the 10% best results
Novelty	2.78	Excellent	In the range of the 10% best results

To quantitatively evaluate the CDM APP, documentation and knowledge facilitation indicators were defined, and seven respondents filled out the questionnaire, assessing them using a Likert scale in an evaluation questionnaire. Based on the survey results, all physicians rated the motivation for using the system as the highest score, citing the possibility of receiving clinical needs through a highly‐rated application. Moreover, all physicians rated the use of the system for easy access to the opinions of other experts as very good. Eighty‐five percent of physicians gave very good ratings to the system's ease of use, citing its appearance, comprehensive coverage of necessary information over time, consistency in messaging, accessibility to essential information, assistance in reducing errors throughout the day, and usefulness for sharing information with colleagues in hospitals. Additionally, 71% of physicians gave a very good rating to the accessibility of necessary information at any time, motivation to use the system for accessing information, usefulness of the system compared to the time spent using it throughout the day, coordination of information elements based on information requirements, practicality of these elements, sufficiency and relevance of answers to questions, and responsiveness and accountability of colleagues in responding to questions. Furthermore, 57% of physicians gave a very positive rating to sharing information and knowledge, ease of using the search function and accessing the system, user control and monitoring, free access to the system, and support from colleagues and system users. They also rated the usefulness and appropriateness of notifications, expressed intent to continue using the system, and the ability to access evidence‐based information as very good. Three physicians expressed no opinion regarding the possibility of allowing other colleagues to access information when necessary and editing information. No bad or very bad feedback was provided in response to any of the questions.

## 4 DISCUSSION

The study has demonstrated that accurate and prompt sharing of information is crucial in mitigating emergency situations, underscoring the significance of requirements engineering. Furthermore, it is imperative to consider the legal requirements and regulations associated with emergency response applications during app design to ensure compliance.^36^, ^37^, ^38^ Notably, 71% of physicians affirmed that CDM app provided access to requisite information at all times. While we have thoroughly evaluated the technical requirements of our app, it is recommended that future studies also examine the legal aspects.

The results of other study indicate that M‐health has a significant impact on improving patient care in the Iranian healthcare system.^39^, ^40^, ^41^ Experts in focus groups emphasized the importance of sharing information accurately and quickly, which can be addressed by developing mobile‐based applications that allow real‐time information sharing among healthcare professionals.

CDM and JOIN are both mobile‐based applications that enable real‐time information sharing among healthcare professionals. JOIN is designed specifically to improve care for acute stroke patients. Both apps facilitate communication between healthcare providers. JOIN is an app that enables communication between healthcare providers. While JOIN's video chat feature allows for better patient assessment, CDM emphasizes communication among healthcare providers, making it more suitable for our research goals. The choice of Property of the app depends on the objectives and priorities of the healthcare organization.^42^, ^43^, ^44^

The integration of technological systems in the healthcare sector has become increasingly crucial for facilitating communication and enhancing patient outcomes. Various applications have been developed to enable the sharing of patient information, catering to diverse requirements. For example, the HELP system emphasizes patient engagement in information sharing, while CDM prioritizes communication among healthcare providers. These contrasting approaches reflect distinct objectives and priorities.^45^

While previous research has mainly utilized the React Native framework for developing mobile applications to evaluate pelvic pain, our study differs by utilizing the Flutter framework with Dart language to develop our remote medical service, CDM.^46^ The decision to use Flutter was based on its numerous advantages over React Native, including its faster development time, improved performance, and cross‐platform compatibility. Our findings suggest that utilizing Flutter may be a promising approach for developing mobile medical services, particularly in remote healthcare settings where access to specialized care can be limited.

As an example of a mobile application built on the Dart‐based Flutter framework, SafeSenora empowers healthcare professionals to continuously monitor their patients' health status, including physicians and nurses. With its cross‐platform compatibility, SafeSenora delivers faster speed, greater efficiency, and esthetically pleasing designs.^47^ In addition, the framework simplifies access to multiple APIs, facilitating system development. It's worth noting that we also utilized Flutter to develop our CDM app.

A comparable technology stack was employed to create telemedicine software for medical image representation, visual synchronization, and collaborative treatment planning. The use of Node.js and Socket.io libraries facilitated bidirectional real‐time communication between servers and clients.^48^, ^49^, ^50^ This software allowed physicians to share medical data instantly, providing immediate visual feedback for collaborative treatment planning, distributed treatment, and remote clinical care programs. It's worth noting that we also utilized Node.js for the back‐end development of our CDM app. The messaging application operates in real‐time, resulting in frequent updates and changes. When developing such an application, Node.js is an ideal choice due to its proficiency in managing heavy user traffic, lightweight design, and rapid message delivery. Thus, the development of messaging apps utilizing Node.js is recommended.

The study found that chat applications developed using Node.js, Socket.io, and MongoDB showed a sixfold increase in performance speed compared to those built with PHP and MySQL. Even when handling high volumes of data, these applications had response times of less than 1 s, making them particularly beneficial for dealing with larger datasets. In fact, Node.js outperforms plain PHP by over 35 times and is more efficient in terms of RAM usage.^48^ As CDM is a real‐time application, it requires the least response time. Therefore, we chose MongoDB.

Effective information sharing is a critical factor in determining the usability of an application. Studies show that higher‐quality information leads to greater user satisfaction.^36^, ^45^, ^47^ The results of the UEQ analysis indicate that the CDM app exhibits high efficiency with a score of 2.40, as well as quality exchange and transparency with a score of 2.9, thereby indicating the provision of high‐quality information.

In previous research, the initial implementation of program has shown that it possesses a user‐friendly interface. Interviews suggest high acceptance rates, and user behavior observations demonstrate that users are proficient in gathering information and managing the app with ease. This finding is significant because individuals tend to rely on an emergency system only after becoming accustomed to it.^51^ In our study, we employed the CDM app in an ED, where physicians were able to navigate and utilize all its features effortlessly. Our findings indicated that 85% of doctors confirmed the system's user‐friendliness due to its intuitive design.

In contrast to the study on a mobile‐based educational app for women at risk of endometriosis, where the standard QUIS questionnaire was used to assess usability and user satisfaction, we used UEQ for evaluation purposes in our CDM project.^52^

## 5 CONCLUSION

The CDM app enables real‐time interaction among healthcare providers, facilitating decision‐making in emergency settings. Additionally, remote application‐based decisions have been found to be as accurate as those made with a physical presence, making it a low‐cost and easily implementable telemedicine solution for centers without full‐time specialists. The use of the UEQ approach ensured that the app design incorporated real user needs effectively. During the usability test, physicians who used the app found that it satisfied their expectations, indicating that the CDM system could help them make informed decisions with ease of implementation, simple app flow, and low cost. To optimize workflow and improve interactions among healthcare providers, the use of text and voice messaging, forms, photos, and videos should be evaluated further. In summary, the CDM application represents a comprehensive process involving input from multiple stakeholders to enable collaboration among healthcare providers in fast‐paced and high‐pressure environments. Preliminary evaluations demonstrate its potential to improve patient care and outcomes, making it an attractive option for centers that cannot afford full‐time specialists. In conclusion, the CDM application has the potential to significantly enhance patient care and outcomes in emergency settings.

## AUTHOR CONTRIBUTIONS

**Neda Shams‐Vahdati**: Methodology; resources; software; validation; writing—original draft; writing—review and editing. **Samad Shams Vahdati**: Conceptualization; supervision; validation; writing—review and editing. **Taha Samad‐Soltani**: Conceptualization; funding acquisition; investigation; methodology; project administration; supervision; writing—original draft.

## CONFLICT OF INTEREST STATEMENT

The authors declare no conflict of interest.

## ETHICS STATEMENT

The Research Ethics Committee of the Tabriz University of Medical Science approved the research proposal (Code of Ethics IR.TBZMED.REC.1401.596).

## TRANSPARENCY STATEMENT

The lead author Taha Samad‐Soltani affirms that this manuscript is an honest, accurate, and transparent account of the study being reported; that no important aspects of the study have been omitted; and that any discrepancies from the study as planned (and, if relevant, registered) have been explained.

## Supporting information

Supporting information.

## Data Availability

Data are available on request from the authors.
