# Supplementary figures and images for: Design and evaluation of collaborative decision‐making application for patient care in the emergency department
Source: Health Sci Rep. 2024 Feb 25;7(2):e1931. doi: 10.1002/hsr2.1931 (PMC10895157; doi:10.1002/hsr2.1931)

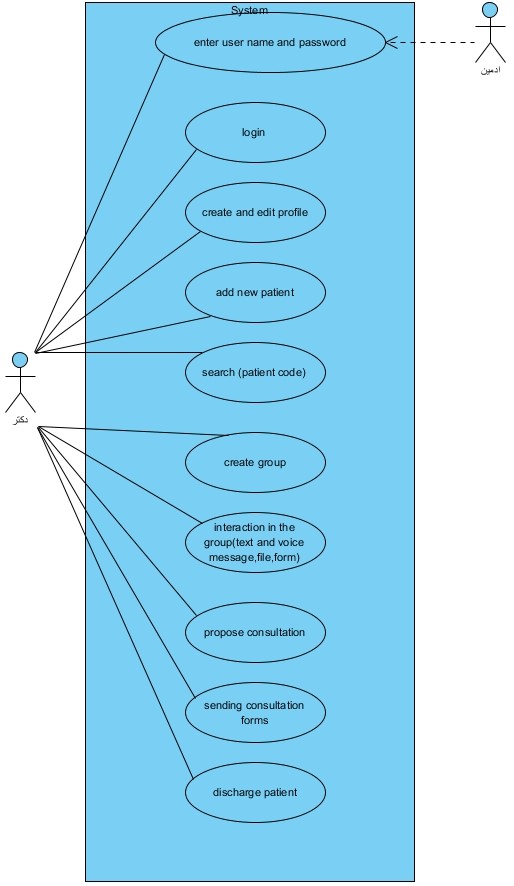

Supplement: Supplementary file 1 — Supporting information. [file HSR2-7-e1931-s001.zip › Appendice1 Use Case diagram of CDM.jpg]

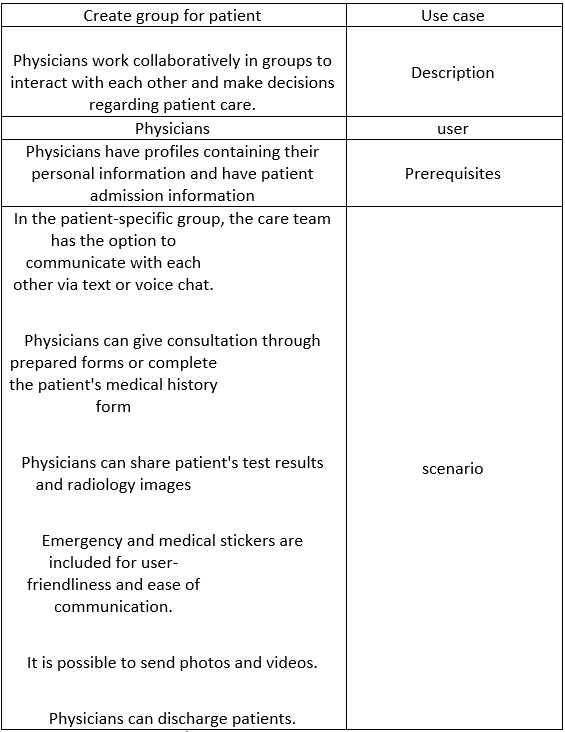

Supplement: Supplementary file 1 — Supporting information. [file HSR2-7-e1931-s001.zip › Appendice2 Create group for patient scenario of CDM.PNG]

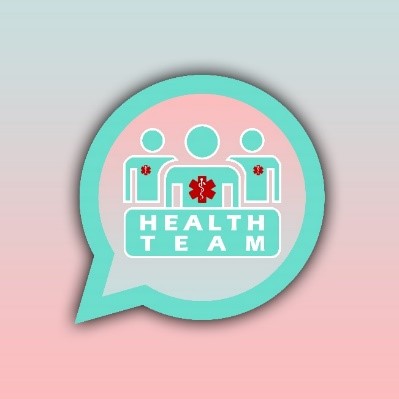

Supplement: Supplementary file 1 — Supporting information. [file HSR2-7-e1931-s001.zip › Appendice3 CDM icon.jpg]

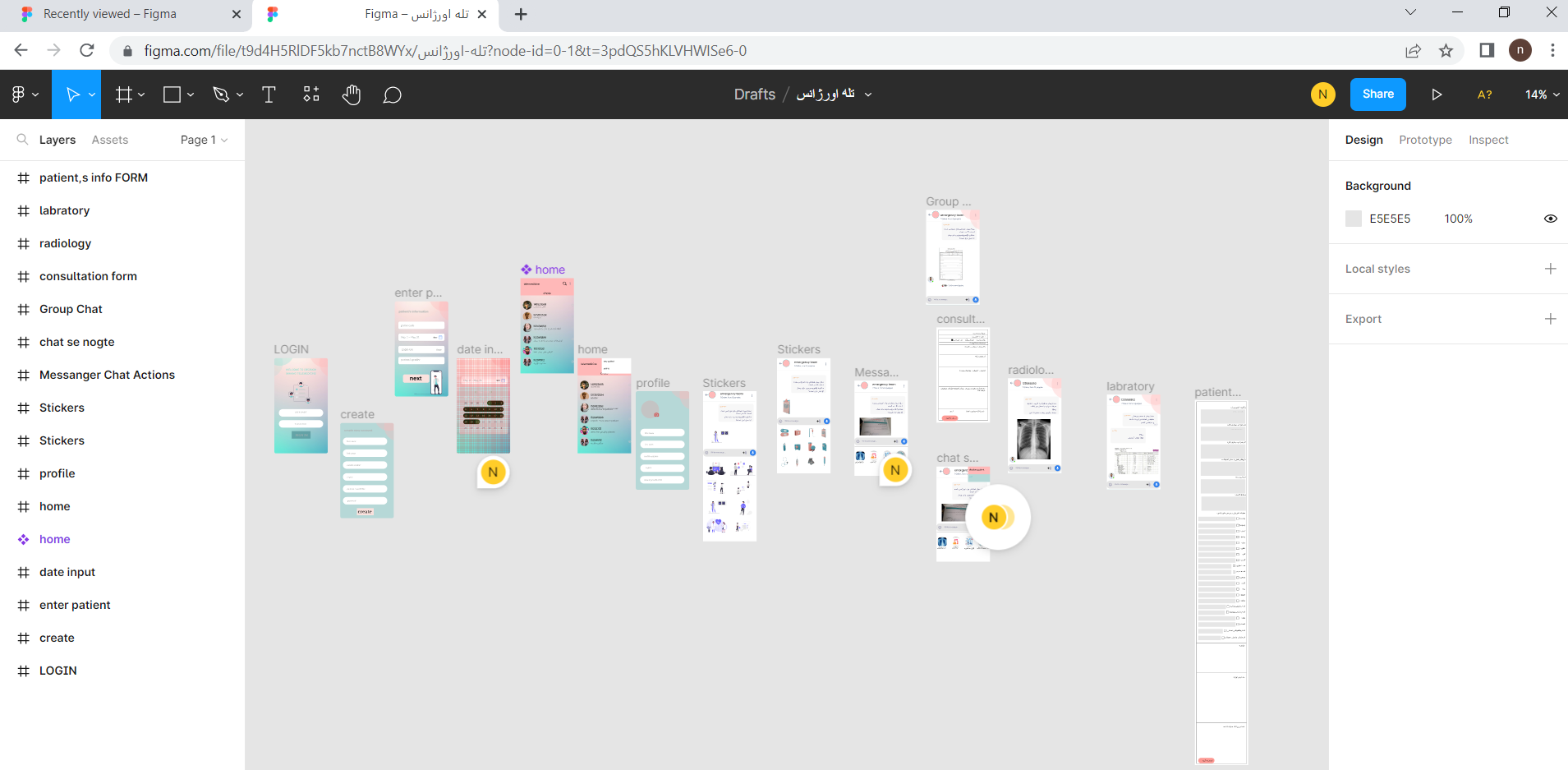

Supplement: Supplementary file 1 — Supporting information. [file HSR2-7-e1931-s001.zip › Appendice4 prototype CDM with figma.png]

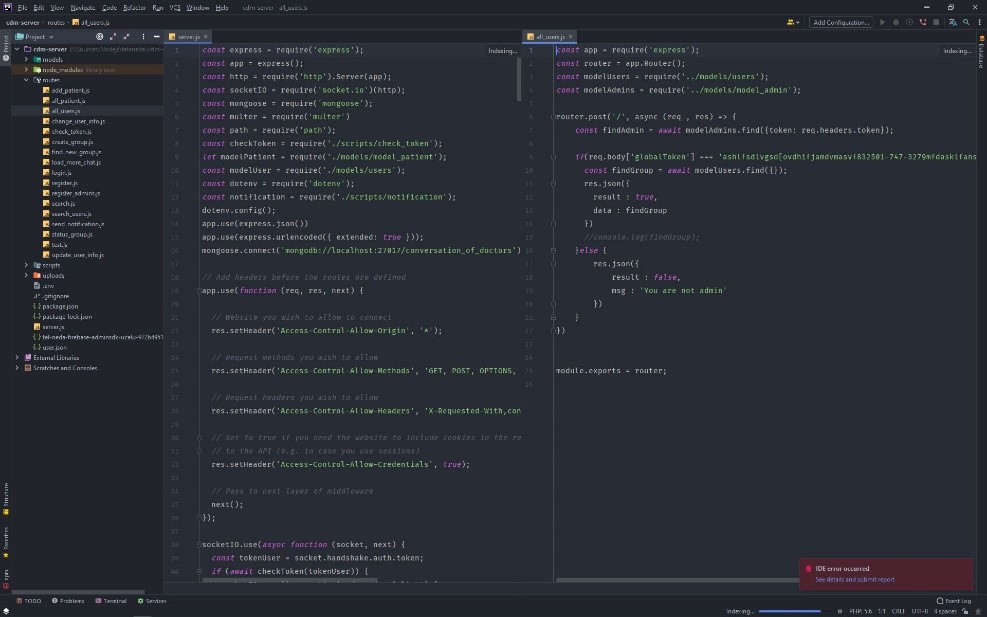

Supplement: Supplementary file 1 — Supporting information. [file HSR2-7-e1931-s001.zip › Appendice5 Node js codes.jpg]

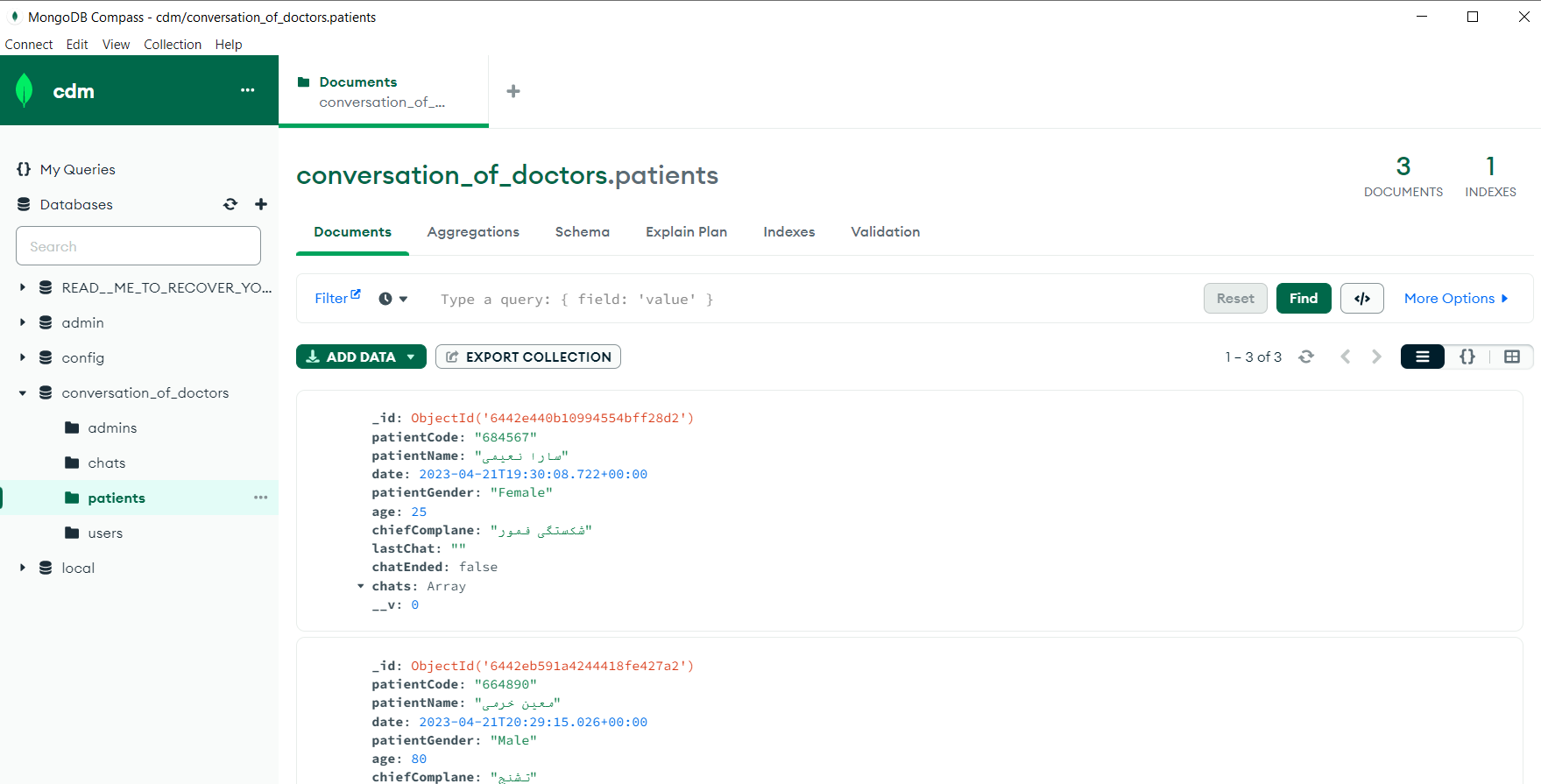

Supplement: Supplementary file 1 — Supporting information. [file HSR2-7-e1931-s001.zip › Appendice6 MongoDB.png]

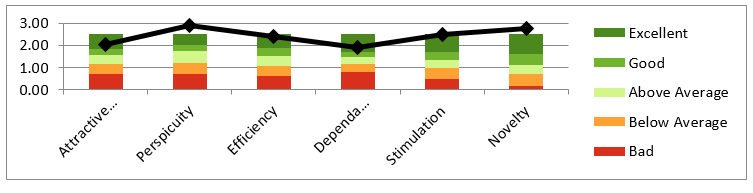

Supplement: Supplementary file 1 — Supporting information. [file HSR2-7-e1931-s001.zip › Appendice9 Benchmark graph for a hypothetical product..PNG]
